# Population sampling affects pseudoreplication

**DOI:** 10.1371/journal.pbio.2007054

**Published:** 2018-10-15

**Authors:** Crispin Y. Jordan

**Affiliations:** School of Biomedical Sciences, University of Edinburgh, Edinburgh, United Kingdom

‘Pseudoreplication’, or the treatment of nonindependent data points as independent, can be difficult to identify [1,2]. Failure to account for pseudoreplication increases the probability of falsely concluding that experimental treatments have real effects (i.e., making a Type I error), and can lead to applying conclusions to the wrong ‘population’ (see paragraph 5). In this light, Lazic and colleagues’ [2] recent article provides a useful resource to identify pseudoreplication when designing and analyzing experiments. However, as illustrated below, discussion of their framework introduces some confusion with respect to sources of pseudoreplication and may therefore misguide some readers for common experimental designs. Lazic and colleagues discuss pseudoreplication through examples that draw from frameworks and examples illustrated in their figures. Therefore, to clarify concepts in [2] this Formal Comment focuses on figures therein.

The heart of the question ‘What is ‘*N*’ (sample size) in my experiment?’ lies in the notion of sample independence. ‘Independence’ implies that measurements are uncorrelated among samples; when correlations exist, analyses must account for them to obtain reliable results. Sample independence can be violated in two ways. First, an experiment’s design can render samples nonindependent. For example, as explained by Lazic and colleagues, applying a treatment simultaneously to a group of subjects (rather than to subjects individually) or interactions between subjects can induce nonindependence. Lazic and colleagues focus on this first cause of nonindependence, and they provide their Fig 3 to help assess independence within an experiment.

Second, nonindependence can arise due to how a population is sampled, which Lazic and colleagues discuss less directly than they do the first source of nonindependence. To illustrate how sampling an entity of interest (the ‘population’) can generate nonindependence, imagine a study that aims to estimate the average height of people in a city. Consider two approaches to obtain height measurements (the ‘samples’). First, we might number all of the families (defined in some convenient way) in the city, randomly select 10 families, and measure the heights of 10 random people per family. We know that family members are likely more similar in height than any two randomly selected individuals from the city because family members likely share similar environments (e.g., similar diets) and (by definition) share genetics. In other words, height measurements are likely to be correlated and are therefore nonindependent due to shared environments or genetics. As a result, the variance in this data set will not properly reflect the city’s true variance in height: variance in height will be lower than the true value for the city, and standard errors (SEs) for the height estimate will be artificially small. Second, we could randomly sample 100 individuals in the city to measure. These measurements will lack correlation (they are independent), and the sample variance will correctly reflect the variance in the population and yield reliable SE.

How can ignoring the details of sampling affect analyses and conclusions? Consider Lazic and colleagues’ Fig 2C, which illustrates an experiment that applies a different treatment to each of a mouse’s eyes for a series of mice, e.g., Treatments A and B may be applied to the left and right eyes, respectively, of the first mouse, B and A to the left and right eyes, respectively, of the second mouse, and so on. Lazic and colleagues argue that each eye represents an individual sample, i.e., the *N* = 1 for each eye, so for four mice, we have *N* = 8. This view suggests using a two-sample *t* test to test for differences between the treatments. However, following arguments above, eyes from individual mice are not independent; a mouse’s two eyes share genetics and their developmental environment, so we expect measurements made on pairs of eyes to be correlated. This recognition of nonindependence suggests the use of a paired *t* test to analyze these data. Simulations (see S1 Text) illustrate how these different perspectives affect conclusions. When between-mouse variance is low (i.e., mice are similar to one another), two-sample and paired *t* tests perform similarly, as they have similar Power and Type 1 error rates. However, when between-mouse variance is comparable to variance in the treatment effect, a two-sample *t* test is vastly underpowered compared to a paired *t* test. Hence, failure to account for nonindependence can lead to loss of power. Of course, this principle also applies to more sophisticated scenarios; for example, a study of gene expression from, say, six tissues, each measured once per mouse, should similarly account for nonindependence within mice.

Fig 2D provides a more concerning case. Here, Lazic and colleagues consider an experiment in which individual mice experience and are measured for a response to a treatment more than once (compared to Fig 2C, in which each treatment is applied to each mouse only once). For example, Treatments A and B are both applied to individual mice twice over the course of an experiment, and a measurement follows each application. Lazic and colleagues argue that each of these four measurements per mouse constitute independent measurements, i.e., *N* = 4 per mouse. Again, the arguments above suggest that the four measurements per mouse are not independent: two measurements made with respect to Treatment A for a focal mouse will be similar because they come from the same treatment but also because they share the mouse’s genetics, age, environment, etc. However, unlike the case in Fig 2C, failing to account for nonindependence here (2D) can increase the Type 1 error rate, if the experiment aims to understand a population larger than a focal mouse (see bottom of this paragraph). To make this point explicit, imagine that we used ANOVA to analyze the experiment in Fig 2D (although the argument applies more generally). Broadly speaking, ANOVA quantifies and compares variance due to treatment effects and residual ‘error’; with correlated samples, their increased similarity decreases the estimate of error variance, thereby artificially inflating a signal of treatment effects and increasing the risk that researchers falsely conclude that a treatment affects their data. These principles apply more generally to experiments in which individuals are repeatedly measured (i.e., beyond experiments with a ‘time’ component; see [3] for additional discussion of time effects), including the use of technical replicates, which are repeated measurements of some entity. (Lazic and colleagues make similar arguments with their ‘nonhuman primate neuron’ example but do not explain how they apply to their Fig 2D.) Note, however, that Lazic and colleagues correctly assert in Fig 2D that each measurement on an individual is independent if the experiment aims to understand treatment effects for that mouse only [2] because each measurement may be viewed as an independent sample from a population of possible responses by that focal mouse. Similarly, if a study uses only samples from a single individual, genotype, or family, it will be pseudoreplicated if it aims to make inferences at a higher level (e.g., for a population or species) but not if the study aims to understand responses only at the level at which they are measured: whether a study is pseudoreplicated depends on its scope.

To conclude, we note that identifying your ‘*N*’ does not in itself aid an analysis when this identification is unconnected to the analysis. In some cases, Lazic and colleagues’ framework to identify ‘*N*’ and their suggestion to simply use an average value per ‘experimental unit (EU)’ in analyses will not suffice. Consider their example experiment that involves randomising multiple body parts from individual animals to treatments. Lazic and colleagues assert that ‘*N*’ equals the total number of body parts. However, this approach does not resolve nonindependence that arises when multiple animals each provide multiple body parts, and more sophisticated methods are required (e.g., including ‘animal id’ as a ‘random’ effect in a mixed effects model). Overall, the safest approach to deal with pseudoreplication involves careful consideration of sampling, experimental design, and the researcher’s goals [1] and applying statistical analyses that correctly characterise an experiment’s complete design to incorporate variance that underlies ‘*N*’ [4].

## Supporting information

S1 Text R code for simulations. (TXT)

## Abbreviation

SE: standard error

## References

[1] Colegrave N, Ruxton GD. Using biological insight and pragmatism when thinking about pseudoreplication. Trends in Ecology and Evolution. 2018; 1 (33): 28–35. Available from: 10.1016/j.tree.2017.10.007 29122382

[2] Lazic SE, Clarke-Williams CJ, Munafo MR. What exactly is ‘*N*’ in cell culture and animal experiments? PLoS Biol. 2018; 16(4): e2005282 Available from: 10.1371/journal.pbio.2005282 29617358 PMC5902037

[3] Pollitt L, Reece SE, Mideo N, Nussey DH, Colegrave N. The problem of auto-correlation in parasitology. PLoS Pathog. 2012; 8(4): e1002590 Available from: 10.1371/journal.ppat.1002590 22511865 PMC3325192

[4] Schank JC, Koehnie TJ. Pseudoreplication is a pseudoproblem. Journal of comparative psychology. 2009 123: 421–433. 10.1037/a0013579 19929110

